# Elevation pattern of resource allocation in *Picea crassifolia* Kom. and its coupling mechanism with soil factors in Helan Mountains, China

**DOI:** 10.1093/aobpla/plaf058

**Published:** 2025-10-13

**Authors:** Kuan Zhang, Liang Jiao, Ruhong Xue, Peng Zhang, Xin Yuan, Xuge Wang, Qian Li, Zhengdong Guo, Yarong Qing, Le Zhang

**Affiliations:** College of Geography and Environment Science, Northwest Normal University, No. 967, Anning East Road, Lanzhou 730070, China; Key Laboratory of Resource Environment and Sustainable Development of Oasis, Northwest Normal University, Lanzhou, Gansu Province 730070, China; College of Geography and Environment Science, Northwest Normal University, No. 967, Anning East Road, Lanzhou 730070, China; Key Laboratory of Resource Environment and Sustainable Development of Oasis, Northwest Normal University, Lanzhou, Gansu Province 730070, China; College of Geography and Environment Science, Northwest Normal University, No. 967, Anning East Road, Lanzhou 730070, China; Key Laboratory of Resource Environment and Sustainable Development of Oasis, Northwest Normal University, Lanzhou, Gansu Province 730070, China; College of Geography and Environment Science, Northwest Normal University, No. 967, Anning East Road, Lanzhou 730070, China; Key Laboratory of Resource Environment and Sustainable Development of Oasis, Northwest Normal University, Lanzhou, Gansu Province 730070, China; College of Geography and Environment Science, Northwest Normal University, No. 967, Anning East Road, Lanzhou 730070, China; Key Laboratory of Resource Environment and Sustainable Development of Oasis, Northwest Normal University, Lanzhou, Gansu Province 730070, China; College of Geography and Environment Science, Northwest Normal University, No. 967, Anning East Road, Lanzhou 730070, China; Key Laboratory of Resource Environment and Sustainable Development of Oasis, Northwest Normal University, Lanzhou, Gansu Province 730070, China; College of Geography and Environment Science, Northwest Normal University, No. 967, Anning East Road, Lanzhou 730070, China; Key Laboratory of Resource Environment and Sustainable Development of Oasis, Northwest Normal University, Lanzhou, Gansu Province 730070, China; College of Geography and Environment Science, Northwest Normal University, No. 967, Anning East Road, Lanzhou 730070, China; Key Laboratory of Resource Environment and Sustainable Development of Oasis, Northwest Normal University, Lanzhou, Gansu Province 730070, China; College of Geography and Environment Science, Northwest Normal University, No. 967, Anning East Road, Lanzhou 730070, China; Key Laboratory of Resource Environment and Sustainable Development of Oasis, Northwest Normal University, Lanzhou, Gansu Province 730070, China; College of Geography and Environment Science, Northwest Normal University, No. 967, Anning East Road, Lanzhou 730070, China; Key Laboratory of Resource Environment and Sustainable Development of Oasis, Northwest Normal University, Lanzhou, Gansu Province 730070, China; Plants, Ecosystems & Climate

**Keywords:** Qinghai spruce, plant-soil stoichiometric characteristics, elevation pattern, coupling relationships, Helan Mountains

## Abstract

Environmental heterogeneity in soil fertility induced by elevation gradients affects trade-offs in tree survival strategies. In arid and semi-arid ecosystems, especially in subalpine coniferous forests in the northwest of China, tree nutrient partitioning strategies and their interactions with the soil environment need to be further explored in depth. We set up three sample plots at different elevations in Helan Mountains, and collected and measured the C, N, and P contents of all organs and soil samples of to explore the nutrient partitioning of Qinghai spruce (*Picea crassifolia* Kom.) and its coupling with soil. N and P contents in plants and soils showed a synchronized pattern of decreasing and then increasing with elevation, while C only changed synchronously at low to middle elevations. The allocation strategies of different elements among organs varied across the elevation gradient. Plants at middle elevation allocated more N and P to thick roots and less to branches and leaves compared to those at low and high elevations. Plant stoichiometric characteristics have a close response and influence relationship with soil stoichiometric characteristics, as elevation increased, the number of plant organ types that significantly responded to soil nutrient changes increased, and at the same time, and the effect of soil C content on the stoichiometric characteristics of plant showed an enhanced trend. This study elucidates the elevation patterns and driving mechanisms of plant–soil stoichiometric characteristics in montane forest ecosystems, which will contribute to better targeting of forest and tree management and conservation from the perspective of plant nutrient partitioning.

## Teaser

In arid and semi-arid regions, subalpine coniferous forests are highly sensitive to soil conditions. Elevation changes affect both soil nutrients and tree growth strategies. This study tested if Qinghai spruce (Picea crassifolia) employs different nutrient allocation strategies at different elevations in the Helan Mountains. Results showed greater sensitivity to soil environment at higher elevations. At mid-elevations, spruce allocated more N and P to thick roots, optimizing growth. Conversely, at the timberline, it preferentially allocated these nutrients above-ground to cope with harsher conditions.

## Introduction

Plant–soil material cycling and energy flow play an important role in ecosystem stabilization, and plant–soil spatial coupling is a central mechanism for understanding geochemical cycling and ecological functioning in terrestrial ecosystems (Wardle and Peltzer 2007, Lambers et al. 2009). In recent years, despite the many advances in plant–soil stoichiometry in revealing nutrient cycling and plant nutrient strategies within forest ecosystems (Elser et al. 2007, Yang et al. 2025), research on how plants form a tight spatial coupling with soils at the important environmental gradient of elevation and how this coupling regulates nutrient partitioning in response to changes in elevation is still relatively limited (Körner 2007, Xi et al. 2021). Therefore, in-depth exploration of the stoichiometric characteristics of key elements such as carbon (C), nitrogen (N) and phosphorus (P) and their spatial coupling patterns in the plant–soil system at different elevation gradients is of crucial scientific value and practical significance for revealing the effects of elevation on the nutrient dynamics of terrestrial ecosystems, predicting the response of plants to changes in the soil environment, and formulating ecological conservation strategies for different elevation zones (Tang et al. 2018, Liu et al. 2021b).

Plant–soil stoichiometric signatures have different spatial patterns along different environmental gradients (Feng et al. 2024). Based on the vertical climatic differentiation of mountainous regions and the divergent adaptation of the same plant in different environments during its growth process, different elevations often exert different temperatures and precipitation on the growth of plants (Billings 1974). Therefore, the expression of C, N, and P ecological stoichiometries in plant organs is often inconsistent across elevation gradients (Zhang et al. 2018). The C content of *Potentilla anserina* L. leaves in the Qilian Mountains decreased significantly, while the N content showed an upward trend, while the P content was reduced. However, there was no significant change in leaf C content across the elevation gradient on the northern slopes of the Tianshan Mountains, and global montane vegetation had higher plant leaf N and P contents at higher elevations (Körner 1989, Wang and Xu 2024). Soil SOC, TN and TP content decreased with elevation in the northern boreal plateau, but the opposite was true in the southern tropics and subtropics (Liu et al. 2021a). These results suggest regional heterogeneity in plant–soil stoichiometry across elevation gradients. However, there is still a lack of research in the arid regions of Northwest China, especially in the Helan Mountains, which is the boundary between monsoon and non-monsoon regions and a key ecological barrier in Northwest China (Jiahui et al. 2023, Shen et al. 2024a). The study of stoichiometric patterns in this region is important for a deeper understanding of its material cycling mechanism and the implementation of effective ecological conservation measures.

Allocation strategies for limited plant nutrients, a key mechanism for plant response to environmental change, reflect evolutionary adaptations and ecological interactions, and balance multiple functional requirements (Zhao et al. 2020, Rehling et al. 2021). To maximize growth and maintain optimal metabolic activity, plants need to balance nutrient partitioning between organs under different stresses. N and P concentrations were higher in leaves than in trunks and roots of shrub biomes in the Hengduan Mountains (Li et al. 2024). *Faxon fir* in the Wolong Nature Reserve in China at elevations of 2800–3600 m also allocate more N and P elements to the leaves at higher elevations (Pan et al. 2024). The trade-offs between plant organ chemometric traits reflect the regulatory strategies of plants in acquiring resources and allocating nutrients in different habitats. Investigating the trade-offs between plant organ chemotaxonomic features at different elevations can help us better understand plant growth strategies at the elevation scale.

At present, the coupling relationship between plant organ stoichiometric characteristics and environmental factors is has become a hot spot of research by many scholars. N and P availability and N and P uptake capacity in plants depend largely on various soil variables (Schachtman et al. 1998). Soil TN increased with elevation in *Quercus serrata* in the Barangay Mountains of southwestern China, but there was no clear elevation pattern for N concentrations in plant organs, but P content in all plant organs and soil TP decreased with elevation (Wang et al. 2018). Different studies have revealed that plant stoichiometric characteristics are influenced by soil factors, and this effect varies according to conditions such as elevation and region, and plants show different adaptations according to changes in the soil environment. Therefore, relevant studies can help us to better understand the coupling relationship between plant tissues and soil nutrients in a specific region.

Helan Mountain is an important ecological barrier in the arid and semi-arid region of northwest China, and is a mountainous ecosystem with a complete vertical belt spectrum (Hou et al. 2022). The unique climatic characteristics and complex topographic features lead to significant differences in temperature and precipitation along the latitudinal gradient, thus providing an ideal natural laboratory for studying the nutritional characteristics of plant organs in response to soil and climate changes. Qinghai spruce (*Picea crassifolia* Kom.) is the most typical vegetation type in the Helan Mountains and has important ecological functions. Studying the relationship between its stoichiometric properties and soil environment can provide a more comprehensive understanding of the plant–soil nutrient dynamic balance and the balanced nutrient allocation strategy of plant organs in different regions. Currently, most of the studies on the Helan Mountains focus on the stoichiometric properties of a single site and a single plant organ, or are limited to the stoichiometric properties of soil at a single site (Gao et al. 2023, Shen et al. 2024b). However, studies on the stoichiometric properties of plant organs and their interactions with soil factors at different elevations are still insufficient. Therefore, in this study, the organ traits (C, N, P, and their ratios) of Helan Mountain soil and Qinghai spruce at different elevations and the coupling relationship between them were investigated with Helan Mountain as the research object.

Although the elevation patterns of plant nutrient allocation have been well documented, and existing studies consistently demonstrate significant elevation-driven variations in plant nutrient allocation strategies (Ortiz et al. 2023, Wu et al. 2024). However, the following key research gaps remain to be addressed: the potential synchronization between plant stoichiometric characteristics and soil nutrient properties across elevation gradients has been overlooked; furthermore, the interactive relationship between Qinghai spruce and soil during elevation changes warrants further investigation. Therefore, we propose the following hypothesis: (1) The stoichiometric patterns of Qinghai spruce organs—including leaves, branches, trunks, fine roots, and thick roots—along with soil stoichiometry, exhibit distinct elevation-dependent variations, this is due to the close connection between the changes in plant nutrients and those in soil nutrients. (2) Plants may have different distribution strategies for nutrients to their organs at different elevations because of the complex coupling dynamics of stoichiometry between Qinghai spruce and soil environment. (3) There is a close interaction between the stoichiometric characteristics of Qinghai spruce growth and soil.

## Materials and methods

### Study area

**Figure 1.**
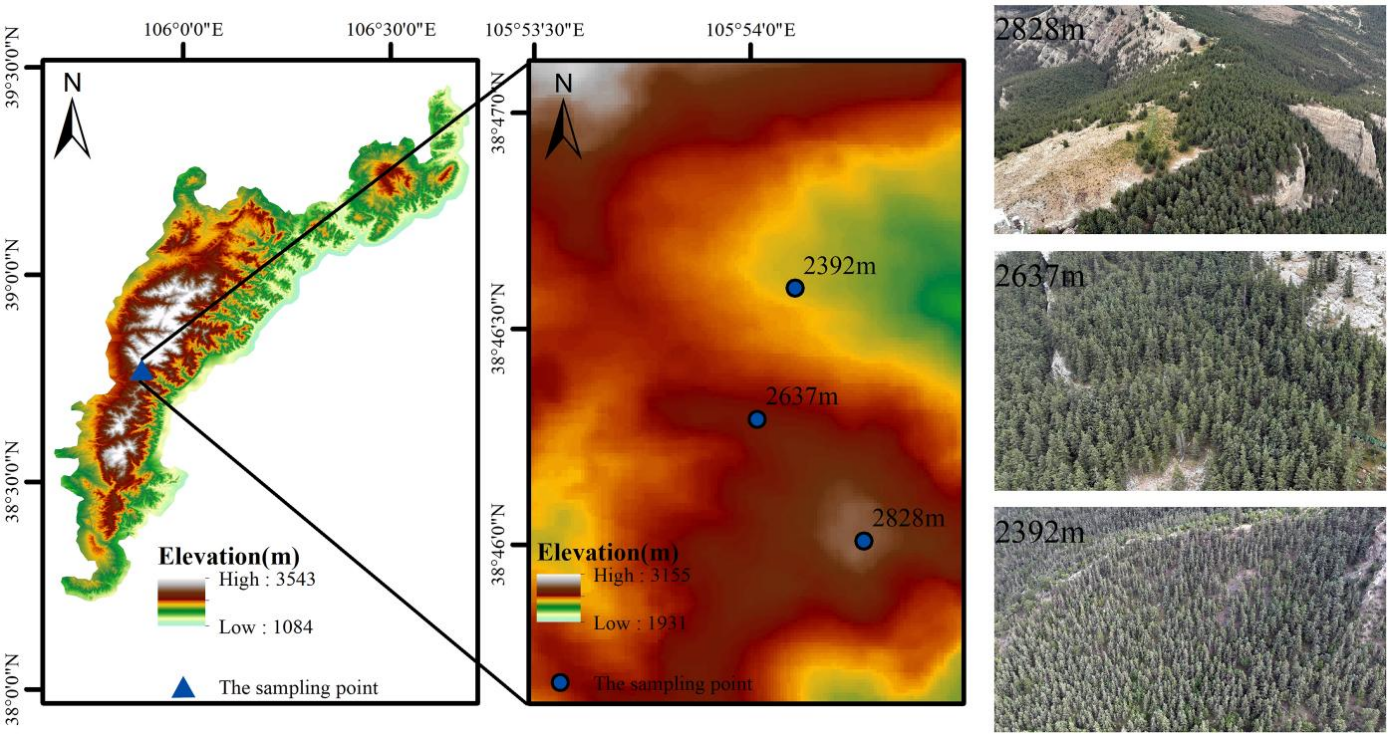
Overview map of the study areas.

The Helan Mountains are located between the Yinchuan Plain and the Alashan Plateau (105°50′E to 106°40′E, 38°10′N to 39°30′N), with a topography that is high in the west and low in the east, and a length of about 140 km from north to south, and an average width of 11.2 km from east to west, with a surface area of about 1820 km^2^, and a main peak elevation of 3556 m (Fig. 1). Its climate is mainly influenced by the Asian summer winds and winter winds (Liu et al. 2005), and is characterized by a mountainous climate, with precipitation most concentrated in June-August, accounting for 60%–80% of the annual precipitation. Qinghai spruce is mainly distributed on the east side of Helan Mountain, between 2350 and 2900 m elevation, and the main growing season is from May to September (Zhao et al. 2018). The mean annual temperature at the foothills of the eastern side of the Helan Mountains is about 8.5°C, and annual rainfall ranges from 202.8 mm in the south to 183.3 mm in the north. According to the Alpine Meteorological Observatory (2901 m, located near the top of the mountain), in the alpine ranges of the mountain, the mean annual temperature is only −0.8°C, but the annual rainfall reaches 429.8 mm. These data show a clear climatic gradient from the foot of the mountain to the summit (Yuan et al. 2000).

### Soil and plant sampling and analyses

Based on the distribution of Qinghai spruce in the Helan Mountains, we set up three sample plots near the upper and lower forest limits and in the intermediate area between them in mid-July 2023, with one 10 m × 10 m sampling area each at each of the low elevation (2392 m), middle elevation (2637 m), and high elevation (2828 m) sites (Table 1). To minimize the effects of light and differences in needle surface temperature on tree elements, each sampling was conducted at midday (Li et al. 2008). To minimize bias from human disturbance, sampling sites were placed away from human habitats. The sample sites were all located in the same slope direction (north slope), with little difference in slope, and the study area had a single tree species, only Qinghai spruce. There was no competition between tree species. Five upright, healthy, undamaged, non-isolated, and of the same size (age, tree height, and diameter at breast height) of Qinghai spruce were selected at each sample site, and the five trees we chose at each elevation were representative of the trees at that elevation. Leaf, branch, trunk, thick root (diameter > 5 mm) and thin root (diameter < 2 mm) samples were collected. Leaf and branch samples were taken from unshaded, mature branches of trees approximately 2 years old and oriented upslope. Trunk samples were obtained by drilling 1.3 m diameter growth cones at breast height and five tree cores were used as trunk samples. Fine root samples were obtained by excavating the soil at a depth of 0–40 cm. Three cores were drilled at the base of each sample tree, i.e. the thick root samples with the highest branching class, using growth cones (Jiao et al. 2019). Soil samples were collected at 0–10 cm, 10–20 cm, and 20–40 cm from 2 m from the trunk of each target tree. Soil samples were collected from three directions from the target trees and the samples were mixed (Liu et al. 2021b). Soil samples were collected partly for measurement of soil bulk density (SBD), soil pH and partly for measurement of soil chemometric properties. After recording the fresh weight of the soil material, the soil samples used for SBD measurement were dried in an oven at 75°C for 48 hours and then the dry weight of the soil samples was recorded.

**Table 1.** The information of sampling point information.

Sampling point	Elevation (m)	Longitude	Latitude
High elevation	2828	105°54′15.76″E	38°46′00.47″N
Middle elevation	2637	105°54′00.95″E	38°46′17.37″N
Low elevation	2392	105°54′06.21″E	38°46′35.62″N

All plant samples were oven dried at 105°C for 30 min on the day of collection and then dried to constant weight at 65°C. Samples were ground through a 0.15 mm sieve using a hybrid ball mill (MM400, Retsch, Germany) and sealed for cold storage (Zhu et al. 2020). Soil samples for soil chemistry measurements were naturally air-dried in the laboratory. They were handpicked to remove vegetation and debris and the samples were completely sieved through a 0.15 mm sieve and sealed for cryogenic storage for soil physicochemical analysis. The plant organic C (C) and soil organic C (SOC) contents of Qinghai spruce was analyzed by K_2_Cr_2_O_7_− H2SO_4_ of external heating method. N and P of plant (N, P) and soil samples (TN, TP) were determined by a fully automated chemical analyzer (Smartchem 200, Advanced Monolithic Sy trunks, Graz, Italy) following a heated digestion method. Soil pH was determined with a pH meter in a soil/water (1:2.5, w/v) suspension. SBD was determined by the soil corer method (Zhang et al. 2018).

### Data analyses

The empirical data were statistically analyzed using SPSS 22.0 software (SPSS Inc., Chicago, IL, United States). One-way analysis of variance (ANOVA) and least significant difference (LSD) tests were used to compare the significant differences in cycad spruce and soil chemometric traits at different elevations (three levels), for each elevation, the average value of five samples of Qinghai fir and five samples of soil from each soil layer was used to represent the value for that elevation. Plant organ traits and soil nutrient contents were expressed as g/kg (based on dry mass), and all C:N, C:P, and N:P ratios in soil and plant organs were measured based on mass ratios. Random forest is a proposed synthesis algorithm based on categorical regression trees (Breiman 2001). By random sampling and increasing the randomness of split variables, the independence between trees in random forests is enhanced and the upper limit of generalization error is reduced. Relative importance was used to measure the effects of soil environmental factors (SWC, SBD, pH, and SOC, TN, and TP) on whole-plant ecological chemometric traits of Qinghai spruce, and the ‘Random Forests’ program package was used to use relative importance in R 4.4.2. The CANOCO 5.0 software was used to study the relationship between the response of C, N, P, and their ratios of each organ of Qinghai spruce to environmental factors using redundancy analysis (RDA), and to investigate in depth the spatial variation pattern of the ecological chemometric characteristics of each organ of Qinghai spruce and its main influencing factors. Graphs were drawn by Origin 2022 software.

## Results

### Elevation patterns of soil stoichiometric characteristics

**Figure 2.**
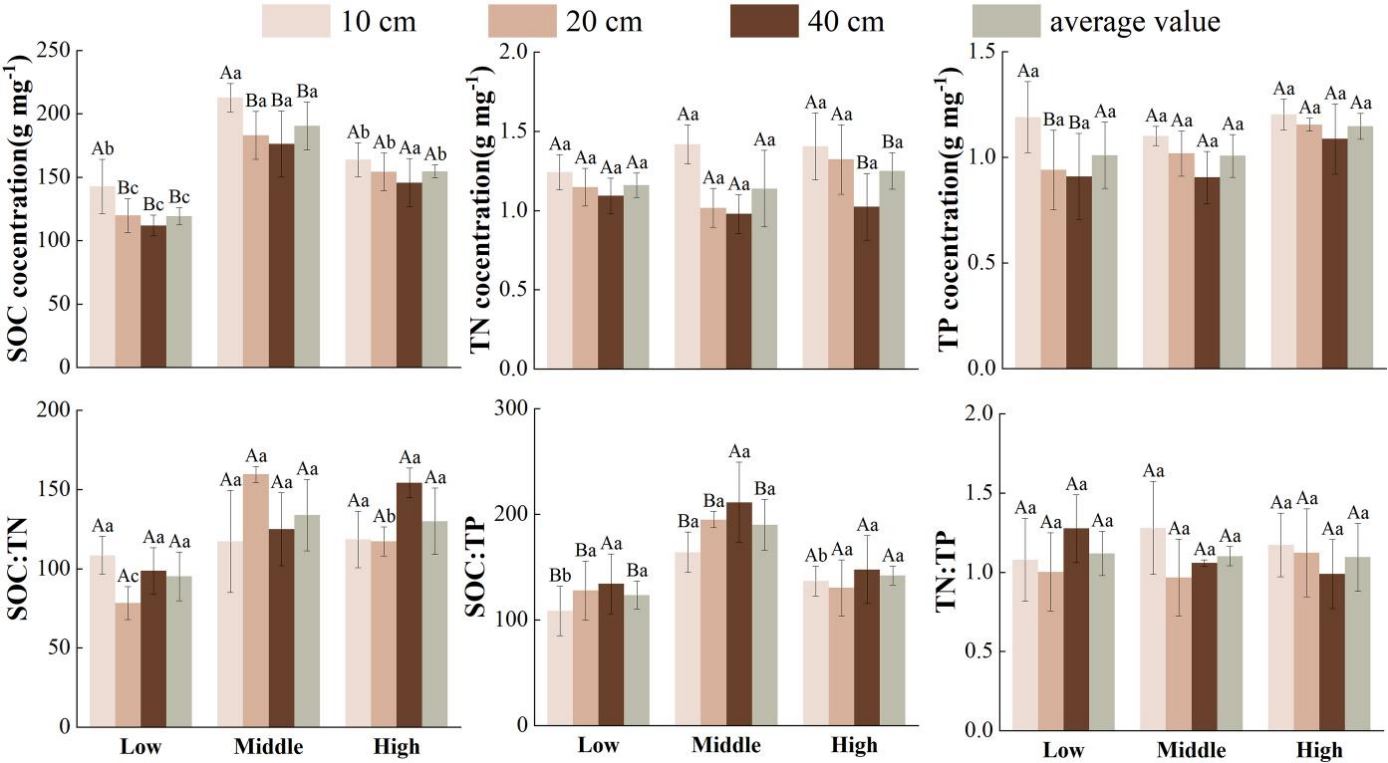
Soil stoichiometric characteristics with soil depths across three elevation levels (low, middle, and high) in the Helan Mountains (SOC: soil organic carbon, TN: soil total nitrogen, TP: soil total phosphorus, different capital letters indicate significant differences in stoichiometric characteristics of different soil depths in the same elevation, and different lowercase letters indicate significant differences in stoichiometric characteristics of the same soil depth in different elevations).

Soil SOC, TN, TP contents and their stoichiometric characteristics were significantly different among the three elevations (Fig. 2). Soil SOC, SOC:TN, and SOC:TP showed a tendency to increase and then decrease with elevation (*P* < 0.05), and were significantly higher at middle elevation was significantly higher than that at low elevation or high elevation, while soil TN and TP showed more stable (*P* > 0.05). At soil depth, soil SOC, TN, and TP contents decreased with deepening soil layers. At low and middle elevation, the soil SOC content in the 0–10 cm soil layer was significantly higher than the 10–20 cm,20–40 cm soil SOC content, while the soil TP content showed the same pattern at low elevations (*P* < 0.05).

### Patterns of plant stoichiometric characteristics

#### Elevation pattern variation of plant C, N and P contents and their ratios

**Figure 3.**
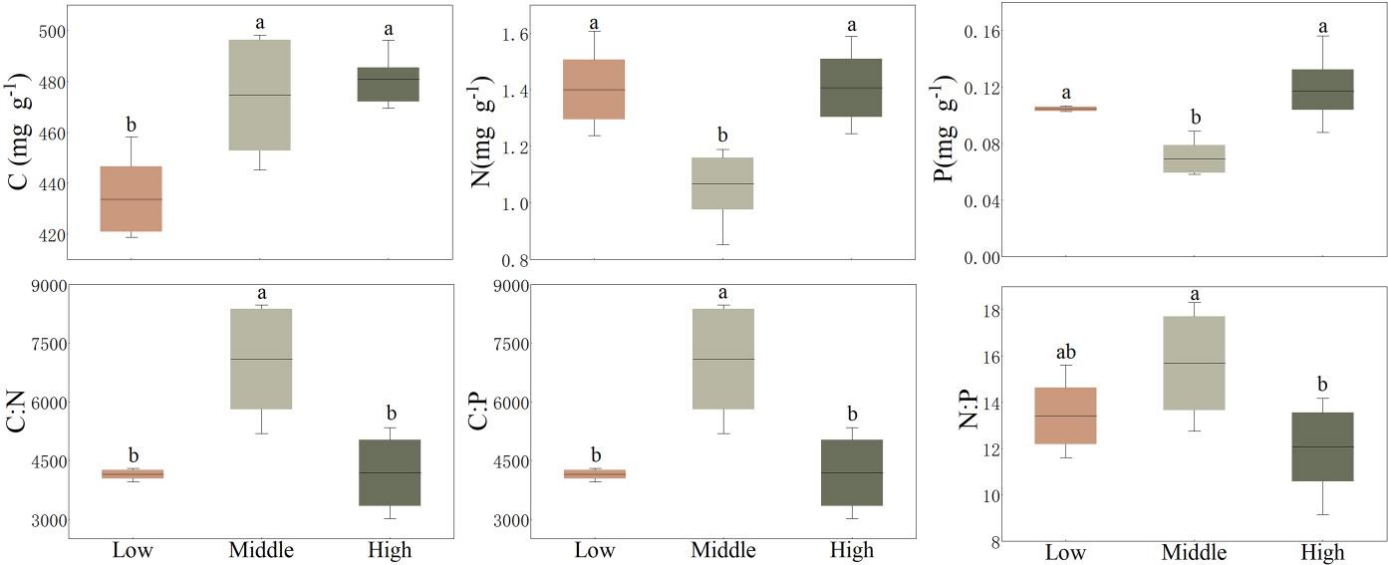
Range for the stoichiometry of whole plant of Qinghai spruce across three elevation levels (low, middle, and high) in the Helan Mountains. Box plots show the 10–90th percentiles with median lines (Different lowercase letters indicate significant differences among elevation levels, *P* < 0.05).

The C, N, and P contents of Qinghai spruce and their stoichiometric characteristics varied greatly at different elevations (Fig. 3). The C content of the whole plant of Qinghai spruce showed an increasing trend with elevation (*P* < 0.05). The N and P contents of the whole plant of Qinghai spruce showed a tendency of decreasing firstly and then increasing later. Notably, the ranking of N and P contents across elevation gradients was: high elevation > low elevation > middle elevation (*P* < 0.05). From low to high elevation, the C:N, C:P, and N:P of the whole plant of Qinghai spruce showed a trend of increasing and then decreasing (*P* < 0.05).

**Figure 4.**
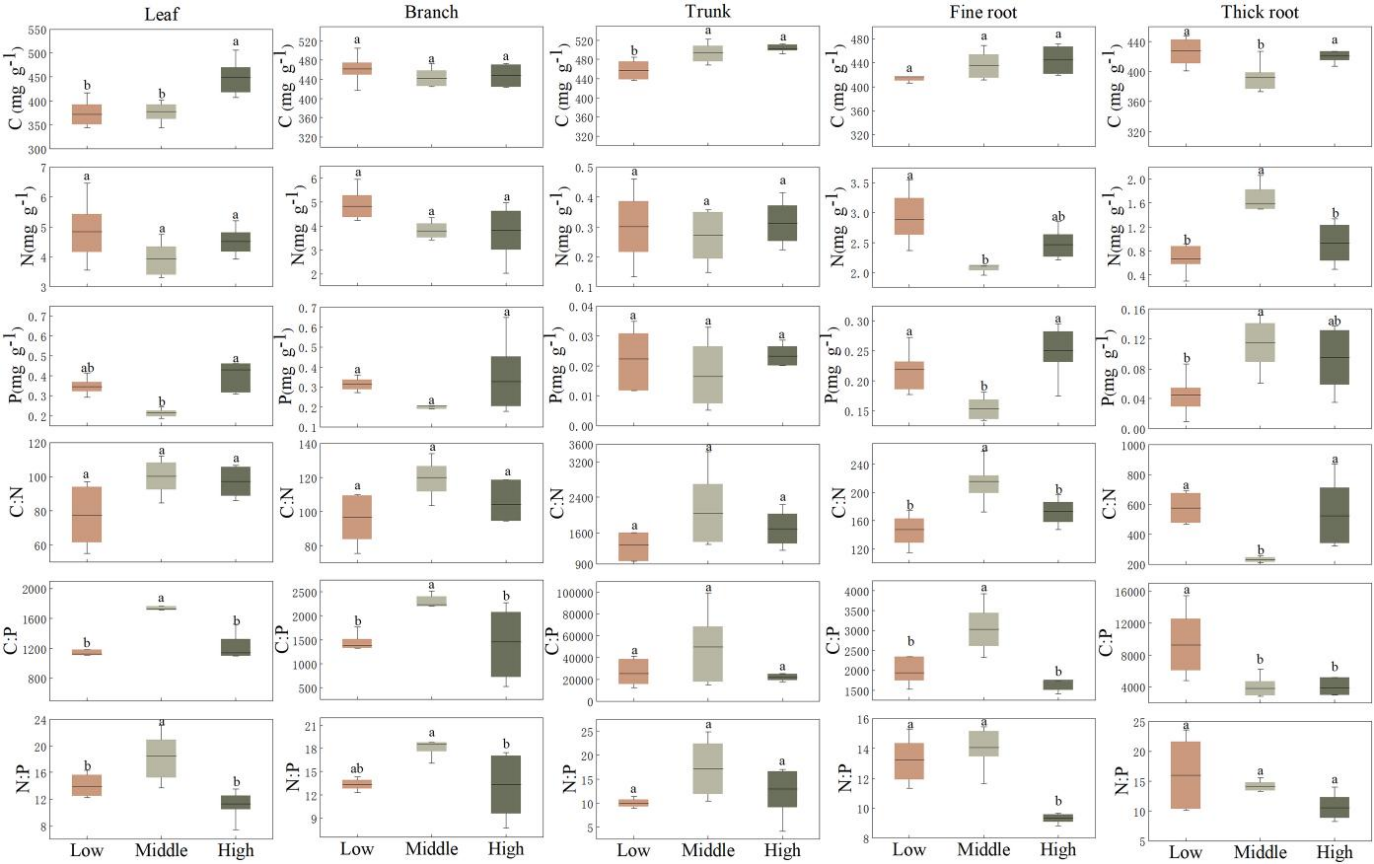
Range for the stoichiometry of Qinghai spruce organs across three elevation levels (low, middle, and high). Box plots show the 10–90th percentiles with median lines. (Different lowercase letters indicate significant differences among elevation levels, *P* < 0.05).

Figure 4 showed the variation of CNP content in different organs of Qinghai spruce at different elevations. C content in leaves, trunk and root showed an increasing trend with elevation, with leaf and trunk C content significantly higher (*P* < 0.05) than that at low elevation. C content in branches and thick roots showed a tendency of decreasing and then increasing, in which the C content of thick roots was significantly higher (*P* < 0.05) than that of low elevation, and N and P content in leaves, branches, trunk and fine roots showed a tendency of decreasing and then increasing with elevation, which was significantly higher (*P* < 0.05) than that of low and middle elevation. In leaves, branches, trunk, and fine roots, C:N, C:P, and N:P all increased and then decreased with elevation.

#### Allocation pattern of nutrients among plant organs

**Figure 5.**
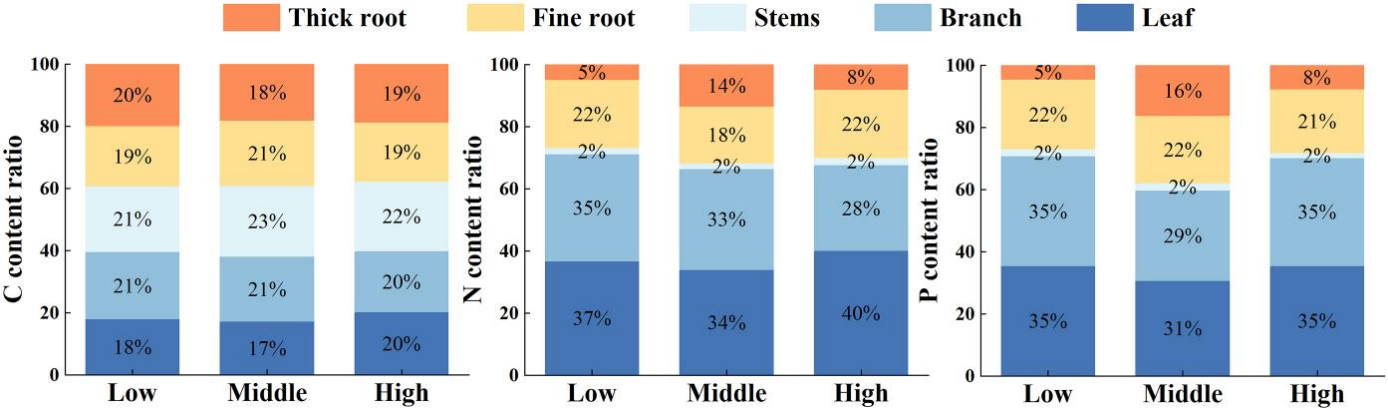
Stoichiometry characteristics of plants as a percentage of each organ across three elevation levels (low, middle, and high) in the Helan Mountains.

The nutrient allocation pattern of plant organs in different regions showed significant differences (*P* < 0.05, Fig. 5). The distribution of C in Qinghai spruce organs did not vary much across elevations, ranging between 18% and 23%. Plant organs at middle elevations allocated more C to the fine roots and trunk, whereas plant organs at low and high elevations distributed it more evenly. The distribution of N and P in Qinghai spruce organs at the three elevations was the largest in the leaves, (low elevation: 37% and 35%, middle elevation: 34% and 31%, and high elevation: 40% and 35%, respectively) and the smallest in the trunks, which were both 2%. The pattern of plant partitioning of N and P was relatively similar at the three elevations, with less being partitioned to the trunk and more to the branches and leaves.

### Explanation rate of soil on plant stoichiometric characteristics

#### Explanation rate of soil on whole plant stoichiometric characteristics

**Figure 6.**
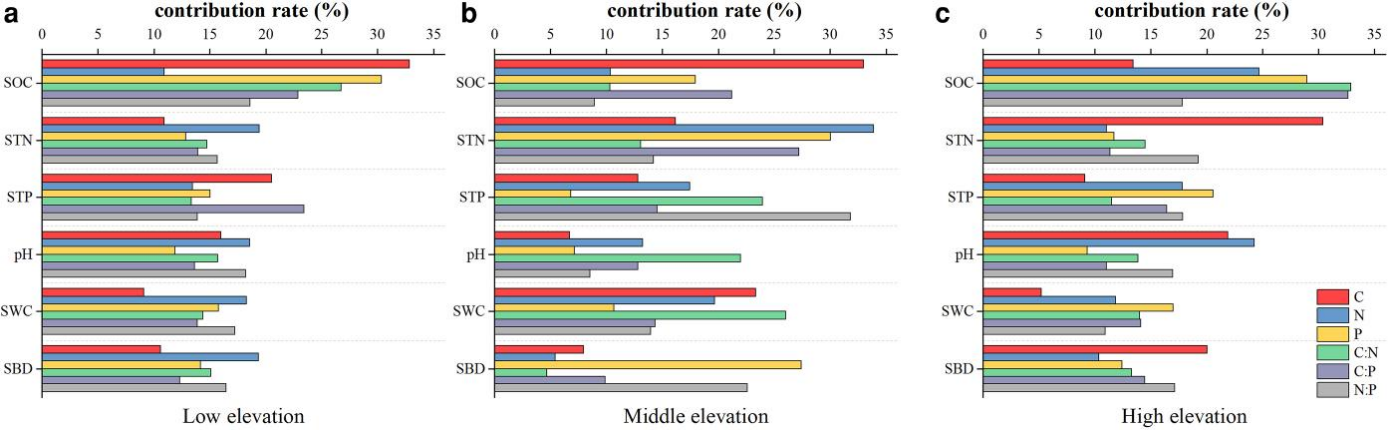
Relative importance of soil environmental factors to Qinghai spruce stoichiometry at different elevations. (SOC: soil organic carbon, STN: soil total nitrogen, STP: soil total phosphorus, SWC: soil water content, SBD: soil bulk density, pH: soil pH).

The results of importance ranking based on random forest showed that different soil factors at different elevations played different roles in plant whole-plant stoichiometry. At low elevation, soil SOC was the most important environmental factor affecting whole plant C, P, C:N and C:P of Qinghai Spruce, with relative importance of 32.85%, 30.33%, 26.73% and 22.89%, respectively. Soil TN mainly affected Qinghai Spruce whole-plant N, with a relative importance of 19.43%. Soil TP mainly affected Qinghai Spruce whole-plant C:P with a relative importance of 23.41% (Fig. 6a). At middle elevation, soil TN was the most important environmental factor affecting the variation of N, P, and C:P of Qinghai Spruce whole plant, with relative importance of 33.89%, 30.01%, and 27.19%, respectively, and soil SOC was the most important environmental factor of C of Qinghai Spruce whole plant, with its relative importance of 33.01%. Qinghai Spruce whole plant C:N mainly Qinghai Spruce whole plant N:P was mainly affected by soil TP, and its relative importance was 31.82% (Fig. 6b). At high elevation, soil C was the most important environmental factor driving whole-plant N, P, C:N, and C:P of Qinghai Spruce, with relative importance of 24.70%, 28.98%, 32.88%, and 32.65%, respectively. Qinghai Spruce whole-plant C and N:P were mainly affected by soil TN, with relative importance of 37.54% and 31.76%, respectively (Fig. 6c).

#### Explanation rate of soil on plant organ stoichiometric characteristics

**Figure 7.**
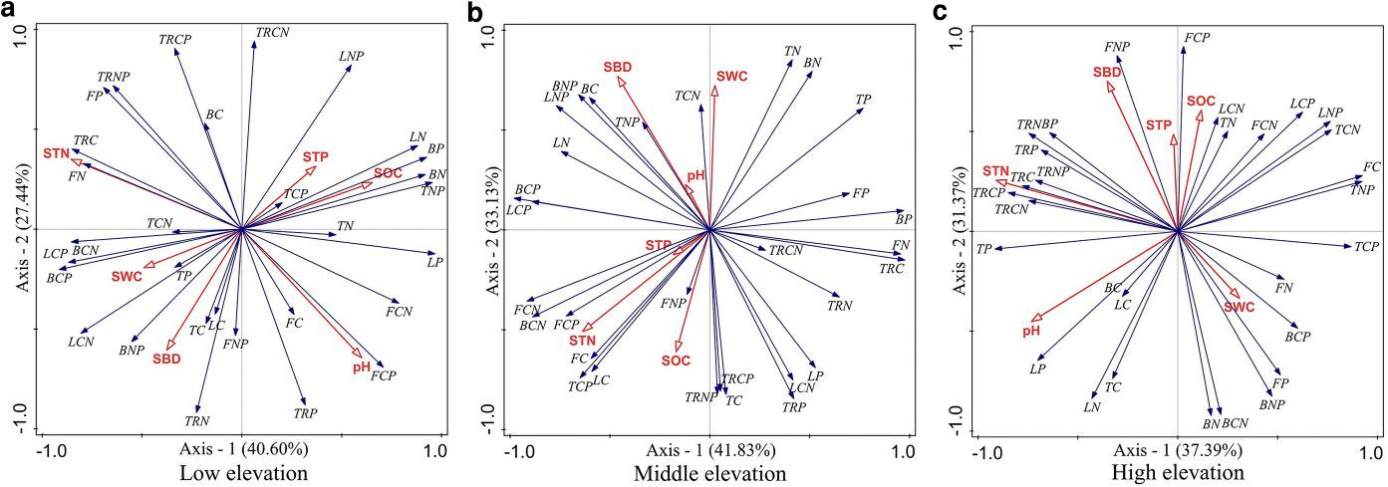
Redundancy analysis of the relationship between stoichiometry in different organs of and soil environmental factors at different elevations (L: leaf, B: branch, T: trunk, TR: thick roots, F: fine roots, S: soil, CN: the C:N ratio, CP: the C:P ratio, and NP: the N:P ratio, SOC: soil organic carbon, STN: soil total nitrogen, STP: soil total phosphorus, SWC: soil water content, SBD: soil bulk density, pH: soil pH).

At low elevation, the results of RDA showed that soil properties elements explained 68.4% (Fig. 7a). Among them, soil TN had the highest contribution of 34.7% to the effect of organ stoichiometry and its ratio in Qinghai Spruce, and there was a significant positive correlation (*P* < 0.05) between soil TN and N and P of fine roots, C and N:P of thick roots, and branch C, and a significant negative correlation (*P* < 0.05) with trunk N. At middle elevation, the results of RDA showed that soil properties elements explained 74.96% (Fig. 7b). Among them, soil TN had the highest contribution to the variation of organ stoichiometry and its ratios in Qinghai Spruce with 28.9%, and soil TN was significantly and positively correlated with C, C:N, C:P of fine roots, leaf C, trunk C:P, and branch C:N (*P* < 0.05), and significantly and negatively correlated with trunk P (*P* < 0.05).At high elevation, the results of RDA showed that soil properties elements explained 68.76% (Fig. 7c). Soil TN had the highest contribution to the variation of organ stoichiometry and its ratio in Qinghai Spruce at 34.1%, followed by soil pH at 29.2%. Soil TN had significant positive correlations (*P* < 0.05) with C, N, P, C:N, C:P, N:P, and P of thick roots, and significant negative correlations (*P* < 0.05) with N of fine roots, and C:P of trunk.

## Discussion

### Elevation patterns of soil and plant nutrient characteristics

In this study, soil C content showed a trend of increasing and then decreasing with elevation, and soil TN and TP content did not show significant differences in its variation with increasing elevation (Fig. 2). These patterns are likely driven by the elevational gradient in climatic and edaphic conditions in the Helan Mountains. At low elevation, higher temperatures and lower precipitation may enhance organic matter decomposition, reducing soil C accumulation (Davidson and Janssens 2006). Conversely, at middle elevation, cooler temperatures and increased precipitation likely promote organic matter accumulation, leading to higher soil C content. At high elevations, colder temperatures and reduced microbial activity may limit decomposition, contributing to the observed decline in soil C (Reich and Oleksyn 2004). The content of soil TN and TP did not show significant differences in its variation with increasing elevation. This might be related to the rate of rock weathering on the eastern slope of Helan Mountains (Liu et al. 2021b, Guoqi et al. 2023). In this study, the soil SOC and soil TN contents showed an obvious decreasing trend with soil depth, and the soil P content remained stable at different soil depths (Fig. 2). This is because soil organic carbon mainly comes from organic matter produced by the decomposition of soil animals and their feces, microorganisms and plant residues, and the content of organic carbon in the surface soil is much higher than that in the deeper soil layer, so soil organic carbon showed a decreasing trend with increasing soil depth. With the increase of soil depth, soil organic matter input capacity soil organic matter and SOC content gradually decreased (Elser et al. 2010, Xue et al. 2016a). Whereas soil P in addition to external organic matter input, weathering of rocks will continuously replenish soil P content so that its content remains stable at different soil depths (Liu et al. 2012).

In this study, Qinghai Spruce C content in the whole plant as well as in the organs leaf, trunk and fine roots were shown to increase with elevation, and Qinghai Spruce N and P content in the whole plant as well as in the organs except thick roots were shown to decrease first and then increase with elevation (Fig. 3). For C content, elevated CO₂ assimilation at higher elevations compensates for reduced photosynthetic efficiency under low temperatures, leading to greater non-structural carbohydrate (NSC) accumulation as cryoprotectants (Millard et al. 2007, Hoch and Körner 2012). Low-elevation trees prioritize root C allocation to mitigate drought stress (Lyu et al. 2017, Bai et al. 2019), whereas high-elevation trees redistribute C to leaves and stems to prevent freezing-induced xylem embolism (Hoch et al. 2002). In contrast, Qinghai Spruce N and P contents are lower at middle elevation because of the harsher environments in which the plant survives at low and high elevations (Dalle Fratte et al. 2019). Moreover, the mean annual precipitation increases with elevation and the mean annual temperature decreases with elevation (Chen et al. 2021), so the hydrothermal conditions are relatively best and the efficiency of N and P utilization is best at low and middle elevations. Moreover, at high elevations with relatively low temperatures, plants resist the inhibitory effects of low temperatures on metabolic rates by increasing N and P content in the body (Reich and Oleksyn 2004).

### Characteristics of nutrient distribution patterns among plant organs

Nutrient partitioning in plant organs under different elevation conditions has different partitioning strategies, and the trade-offs between plant organ stoichiometric characteristics reflect the regulatory strategies of plants in acquiring and distributing resources in different habitats. In this study, C was found to be more evenly distributed among organs at all three elevation gradients (Fig. 5). The uniform C distribution across organs reflects an adaptive response to arid conditions at all elevations. While atmospheric CO₂ remains the primary C source, limited soil water availability reduces root absorption efficiency, forcing plants to optimize internal C partitioning rather than relying on rhizosphere resources (Körner 2003, Hartmann et al. 2020). N and P were more distributed in branches, leaves and fine roots at high elevation (Fig. 5). This is because at high elevation the low temperature environment can be stressful to the plant, resulting in slowing down or even stagnation of metabolic activities in the plant whereas at low elevation it is the drought that can be stressful to the plant. In order to adapt to this low temperature and drought environment, plants may allocate more N and P elements to the parts that can produce heat, such as branches, leaves, and fine roots, in order to increase the metabolic activity of these parts and produce more heat to withstand the low temperature (Mayor et al. 2017, Rahman et al. 2020). Compared to low and high elevations, at middle elevation plant N and P content of thick roots was significantly increased, while branch, leaves, and fine roots N and P content was decreased (Fig. 5). This is due to the moderate environmental conditions such as climate and soil in the middle elevation of Helan Mountains. Moderate climatic conditions can reduce the stress of plants in extreme environments, enabling them to utilize nutrient resources more efficiently, which is conducive to the growth and development of the plant's roots, and thus improves the capacity of the thick roots to absorb and store nutrient elements such as N and P. (Liu et al. 2015, Lu et al. 2022). Compared with the more demanding habitat of the timberline area, Qinghai spruce showed a more balanced and optimized nutrient allocation strategy in the relatively suitable habitat of the middle elevation area.

### Relationships between plant organ traits and soil nutrients among different elevations

At low elevation, the significant effects of soil SOC on whole-plant C, P, C:N and N:P were reflected in the organ effects on fine roots C, C:N, leaf P, N:P, and trunk N:P, whereas the significant effects of soil TN on whole-plant N of Qinghai Spruce were reflected in the organ effects on fine roots N content. Therefore, at low elevation, the organs that significantly responded to the variation of soil SOC were trunk and fine roots, and the organ that significantly responded to the variation of soil TN was fine roots. Taken together, at low elevation, fine roots were the most sensitive organs to the variation of soil physical and chemical factors, which is consistent with the existing research results (Staszel et al. 2022), and reflective of a broader coniferous trait where fine roots rapidly adjust to soil heterogeneity (Foerster et al. 2021, Xie et al. 2023). At low elevation, where fine roots as the primary sensitive organs, their high turnover and exudation rates can SOC inputs through root litter decomposition and rhizosphere priming, thereby enriching soil TN availability via enhanced microbial activity (Ayres et al. 2009). This root-mediated feedback helps stabilize soil nutrient cycles in relatively nutrient-rich low-elevation environments.

At middle elevation, the significant effect of soil TN on whole plant N, P, and C:P of Qinghai Spruce was reflected in the organ on leaf N, P, and C:P of trunk and fine roots, while the significant effect of soil SOC on whole plant C of Qinghai Spruce was reflected in the organ on C content of trunk and fine roots. Therefore, the organs that significantly responded to soil TN variation at middle elevation were leaves, trunk and fine roots, and the organs that significantly responded to soil SOC variation were trunk and fine roots. Taken together, trunk and fine roots were the most sensitive organs to soil physicochemical factor variation at middle elevation. To enhance elevational adaptability, Qinghai spruce develops increased numbers of sensitive organs with rising elevation. This adaptive strategy finds parallel in *Fagus sylvatica* populations within the Carpathian Mountains, where European beech exhibits progressively strengthened acclimatization mechanisms along elevational gradients (Staszel et al. 2022). At middle elevation, the involvement of trunks and fine roots in responding to soil variations suggests that woody biomass accumulation in trunks contributes to long-term SOC sequestration through recalcitrant lignin inputs, while fine roots continue to facilitate nitrogen mineralization, potentially offsetting soil nutrient depletion under moderate environmental stress (Cornejo et al. 2023).

At high elevation, the significant effect of soil SOC on whole plant N, P, C:N, C:P of Qinghai Spruce was reflected in organs on dry N, C:N, C:P of leaves and roots, whereas the significant effect of soil TN on whole plant C, N:P of Qinghai Spruce was reflected in organs on leaf C, dry N:P, fine roots C and thick roots C, and thick root N:P. Therefore, at high elevation the organs that significantly responded to soil SOC variability were leaves, trunk, and fine roots, and the organs that significantly responded to soil TN variability were leaves, trunk, fine roots, and thick roots. Taken together, at high elevation, leaves, trunks, and fine roots were the most sensitive organs responding to soil physical and chemical factor variability. It has been suggested that Qinghai Spruce at high elevation may be more susceptible to changes in soil stoichiometry (Zhang et al. 2023). At high elevation, the broader sensitivity of leaves, trunks, and fine roots implies multifaceted influences: leaf litterfall from nutrient-adjusted foliage can elevate soil SOC and TN by providing labile organic matter, trunks add structural carbon to the soil profile, and fine roots promote nutrient retention in cold, low-decomposition conditions, mitigating the inhibitory effects of low temperatures on mineralization (Liu et al. 2016, Li et al. 2017).

In this study, it was found that the plant organs sensitive to the soil nutrient environment changed from fine roots at low elevation to trunks and fine roots at middle elevation to leaves, trunks, and fine roots at high elevation, and the plant organs sensitive to the soil nutrient environment became more and more sensitive to changes in the soil nutrient environment with the elevation. This progression reflects elevational eco-physiological adaptation: decreasing temperatures constrain metabolic rates, forcing high-elevation trees to optimize leaf nutrient resorption and trunk storage, while increasing precipitation alters soil leaching patterns, driving multi-organ nutrient acquisition strategies as roots alone become insufficient under combined thermal-hydrological stresses (Körner 2003, Reich and Oleksyn 2004). In this environment, fine roots, as the part of Qinghai Spruce in direct contact with the soil, were the first to sense the changes in soil nutrients and responded by adjusting their growth strategies. With the further increase of environmental pressure, the trunk and leaves of Qinghai Spruce also gradually showed sensitive responses to the changes in the soil nutrient environment, and jointly adapted to the new growth conditions to ensure the survival and reproduction of the plant (Qin et al. 2022, Xie et al. 2023, Xue et al. 2023). As for the effects of soil on plants, soil SOC and TN contents showed significant effects on all organs and occupied an important position among the three elevations. At both low and middle elevations, soil TN was the most important influence factor, with soil SOC coming second. And after reaching high elevation, soil SOC surpassed soil TN as the most important influence factor. This may be because at high elevation, as the temperature decreases, lower temperatures inhibit the mineralization and decomposition of organic matter, thus inhibiting the uptake of N and P by plants. In this case, Qinghai Spruce may need to rely more on soil carbon sources to satisfy its growth and metabolism, because carbon sources may be relatively stable and easy to be utilized at low temperatures, a phenomenon that supports the temperature-biogeochemical hypothesis (Xue et al. 2016b).

However, the relationships between plant organ traits and soil nutrients are not unidirectional; plant traits also exert reciprocal influences on soil nutrient pools, contributing to a dynamic feedback loop that enhances ecosystem resilience along elevational gradients. Overall, as elevation increases and sensitive organs diversify, Qinghai Spruce's traits amplify soil nutrient replenishment, supporting the temperature-biogeochemical hypothesis by fostering adaptive nutrient cycling (Weemstra et al. 2021, Han et al. 2022, Matović et al. 2024). This bidirectional interaction underscores the co-evolutionary dynamics between plants and soils in montane forest (Laganière et al. 2010).

## Conclusions

We found that N and P contents in both plants and soils showed a synchronous pattern of decreasing and then increasing with elevation, whereas C only varied synchronously from low to middle elevations and diverged at high elevations. Our results suggest that plant allocation strategies for different elements among organs differed across the elevation gradient, with plants preferring to store N and P in thick roots and displaying a more balanced and optimized allocation strategy at middle elevation, a relatively hospitable habitat, compared to low and high elevations, which are the more demanding habitats of the timberline region. In addition, the results indicated that plant stoichiometric traits were closely related to soil stoichiometric traits in terms of response and influence, with an increasing number of plant organ types significantly responding to soil nutrient changes with elevation, as well as an enhanced trend in the influence of soil SOC on plant stoichiometric traits. This study explains the plant nutrient partitioning strategies and their interactions with soil stoichiometry at different elevation gradients, and provides a scientific basis for targeted forest and tree care and maintenance.

## Supplementary Material

plaf058_Supplementary_Data

## Data Availability

The raw data and R code supporting the findings of this study are available in the Supplementary materials of this article.
