# Supplementary material for: Elevation pattern of resource allocation in Picea crassifolia Kom. and its coupling mechanism with soil factors in Helan Mountains, China
Source: AoB Plants. 2025 Oct 13;17(6):plaf058. doi: 10.1093/aobpla/plaf058 (PMC12574673; doi:10.1093/aobpla/plaf058)
Supplement: plaf058_Supplementary_Data [file plaf058_supplementary_data.zip › R data.pdf]

```r
pacman::p_load(dplyr,randomForestExplainer,randomForest,stringr,caret,pROC,ggplot2,A3)
rf <- HWQ
rf
set.seed(999)
mytheme<-theme_bw()+theme( axis.title =element_text(size = 10),#plot.title = element_text(lineheight = 0,face="bold"),
                           axis.text =element_text(size=8),
                           panel.grid.major =element_line(color ="white"),
                           panel.grid.minor =element_line(colour = "white"),
                           strip.background =element_rect(color = "black",fill="white"),
                           plot.margin =unit(c(0.5,0.5,0.5,0.5), "cm"),
                           axis.text.x =element_text(size = 10,color = "black"),
                           axis.text.y =element_text(size = 10,color ="black"),
                           legend.text =element_text(size = 10),
                           )

rf_train <- randomForest(Mg~., rf,
                         ntree =1000,
                         mtry = 3,
                         importance = TRUE,localImp = TRUE,
                         proximity = TRUE,na.action=na.omit)

rf_train
importance2 <- importance(rf_train)
importance2 <- data.frame(importance2)
importance2$variable <- row.names(importance2)
importance2

importance <-measure_importance(rf_train)
importance <- data.frame(importance)
importance
 #

  for (variable in rownames(importance)) {
    if (importance[variable,"p_value"] >= 0.05) importance[variable,"sig"] <- ''
    else if (importance[variable,"p_value"] >= 0.01 & importance[variable,"p_value"] < 0.05) importance[variable,"sig"] <- '*'
    else if (importance[variable,"p_value"] >= 0.001 & importance[variable,"p_value"]< 0.01) importance[variable,"sig"] <- '**'
    else if (importance[variable,"p_value"] < 0.001) importance[variable,"sig"] <- '***'
  }
data <- full_join(importance,importance2,by="variable")
data

  p1 <- ggplot(data, aes(x = X.IncMSE, y = reorder(variable,X.IncMSE), labs="trees")) +
    geom_col(fill="gray50",color="black",width = 0.8)+ labs(size="no_of_nodes")+geom_text(data=data, aes(x=X.IncMSE+2, y= variable, label = sig))+
    xlab("Relative importance (%)")+ylab("Variables")+mytheme+guides(fill="none")+
    #scale_x_continuous(expand = c(0, 0), limit = c(0, 40))+
    #scale_fill_gradient(low='red',high='blue') +
    #annotate('text', label = 'Mg', x = 3000, y = 2, size = 4) +
    annotate('text', label = sprintf('italic(R^2) == %.2f', 0.36), x = 32, y = 1, size = 4, parse = TRUE)+
    theme(axis.ticks.length.x=unit(-0.1, "cm"), axis.ticks.y=element_blank() )
    #annotate('text', label = sprintf('italic(P) < %.3f', 0.001), x = 9, y = 12, size = 3, parse = TRUE)

  p1
```
